# Clinical Notes

**Published:** 1884-05

**Authors:** J. T. Kent

**Affiliations:** St. Louis, Mo.


### CLINICAL NOTES.

PROF. J. T. KENT, A. M., M. D., ST. LOUIS.

KALI SULPH.—There is no remedy so competent for rattling in the chest when that state has followed an acute attack of inflammation. When a child has passed through a broncho-pneumonia and seems to have recovered and after every change in the weather to cold the child coughs and rattles in the chest, then it is that this remedy cures.

A boy four years old was brought to my office for treatment. He looked well, but coughed several times with a *rattling cough.* "He never expectorates," says the father, "but he always has that rattling. It is worse in cold weather. He eats well and seems well, but always has more or less rattling."

Kali sulph.$^{200}$, one dose, dry, cured the case. In one week the rattling that had been there all winter was gone. The weather changes do not affect him now.

A little girl baby fourteen months old had a very violent double pneumonia last winter. Having been called to the case rather late, it was with great difficulty that the baby was saved. But, finally, it convalesced and looked well. During the cold spring weather it *rattled* in the chest and coughed. Otherwise it was healthy and plump. Some two months after the acute attack it was rattling when the weather changed to cold or damp.

Kali sulph.$^{200}$ cured immediately.

I prescribe Kali sulph.$^{200}$ for rattling in the chest, with or without much cough, in the absence of distinct indications for other remedies—in *subacute* or *chronic* cases.

A child two years old, plump and well nourished. Ringworms on chest and face.

Dulc., Hell. nig., Nit. ac., Phos., Sepia, Tellur (*Lippe Rep.*).

The child craves meat and refuses everything else, ravenously clawing at the meat-plate, stuffing its mouth full to choking if permitted.

Craves meat: Abies can., Aloes, Aur. met., Ferr. met., Hell. nig., Lil. tig., Mag. c., Menyan., Merc. s., Merc. v., Nat. m., Sabadil., Sulph.

Child keeps up a chewing motion during sleep, grinds its teeth: Ars., Bry., Cicuta, Cina, Hell., Pod.

Child rolls its head during sleep: Hell. and others.

Hell.$^{1000}$, two powders—one at night, the other in the morning, and Sac. lac.

The ringworms disappeared promptly.

Mrs. H., æt. 28, married, came to me for treatment. She had a sore on the red part of the lower lip as large as a hickory nut. It was dry and covered with a dry scab; it was hard as horn; it had been several months forming and was quite painful. The sub-maxillary gland was enlarged and hard. The lymphatics were enlarged on the right side of the neck and she had enlarged tonsils. The history of tuberculosis was in the family and she had been told that this was an epithelioma. Several dry scales had been removed, and as soon as one had separated a new one had formed.

She got *Merc. proto-iod.*$^{100}$ (home-made), one dose every four days. It healed in five weeks—perfectly. I withhold my opinion as to diagnosis. I have neglected to mention that it had been deeply cauterized before calling upon me for treatment.

Mrs. R., a married lady, æt. 36, was taken violently ill with pains in right ovary. It was time for menstrual nisus. Her suffering was very intense and she called her usual allopathic attendant. Morphine hypodermically administered failed to give her the desired relief. She grew worse for four days. Dr. B., one of our medical students—boarding in the house at the time—was asked to try his hand, but he advised them to send for me.

When I arrived her suffering had not abated. The pains were all over the body and the family were fearing a fatal termination, with no confidence in Homœopathy. But allopathy had failed, and something must be done.

She was restless and thirsty. There was sweat and coldness. The pains were even worse in the sweating condition than before. Extremities cold. Fetor of breath and sour perspiration. Lifting of the covers chilled her.

I prepared Merc. sol.$^{6000}$ in water and left the young man to administer it. In two hours she was sleeping soundly—the first rest for four days. No more medicine was required. She took Sac. lac. for a few days and was dismissed.

Mrs. M., æt. 27, married, was taken with violent tearing and stinging in the right ovary. She called a homœopathic physician, who gave her Apis, Lyc., Bell., Lach., but without benefit. When I saw the case she was suffering most intense pain all over the body. There was great thirst, hot perspiration, which did not improve the pain; fetor of the breath, vomiting bile, restlessness, and her screams were heard by the neighbors.

Merc. sol.$^{6000}$, one dose, dry, brought sleep. She had been subject to these attacks, but never had had so violent a one before. She has never had one since.

In looking over the symptoms of these two cases, where can a remedy be found that could cover any part of the case but Merc.? Apis was excluded (although there were stinging pains) by the fact that she must be warmly covered and no relief from perspiration. Where Apis is indicated, patient will throw the covers off; the cool air relieves. The pains did not go from right to left, as in Lycopodium; there was not the heat, burning, throbbing, and aggravation from jarring the bed, like Bell.; there was no lifting of the covering, nor left to right, so peculiar to Lach. But Merc. was the simillimum, and it cured —as the appropriate remedy always cures.

A young man (twenty-eight) called on me to treatment. I found a gleety discharge, entirely painless, gluing the meatus in the morning. He had contracted gonorrhœa several months before, and it had nearly stopped discharging. Five years ago he had an attack of gonorrhœa which resulted in producing a stricture, for which he had been operated on. I could only pass a No. 8 bougie at this time.

The symptoms upon which to base a prescription were: Slight, painless discharge, gluing the meatus; sickly, sallow face; constipation, sour stomach, general debility.

He took Sepia$^{cm}$ (Fincke), one dose dry; then Sac. lac.

The next night he sent a note, saying he was very sick; to please send him medicine; that the discharge had come back.

I sent him Sac. lac., requesting him to come to the office as soon as able.

He called in a few days. He said the medicine sent him gave him great relief. The discharge was profuse, thick, and yellow. Sac. lac. was given, and advised to call in a week.

Next call: Discharge yellowish green; some pain on micturition; night-sweats; bone pains, worse during the perspirations. Had a chill during night.

Merc. sol.$^{6000}$ in water every three hours for twenty-four hours and Sac. lac.

One week later: Symptoms all improved; discharge diminished.

Merc. sol.$^{6000}$, one dose; then Sac. lac.

One week later: Discharge nearly gone; feeling well; possesses as large a stream of urine as ever. He took one dose a week of Merc. sol.$^{6000}$.

At the end of three months passed a No. 14 bougie. No discharge. He is in good health. The bougie passed without effort. The two remedies had completely cured the stricture and the treatment was painless. Could anything have been more satisfactory?

